# Tang Luo Ning, a Traditional Chinese Compound Prescription, Ameliorates Schwannopathy of Diabetic Peripheral Neuropathy Rats by Regulating Mitochondrial Dynamics *In Vivo* and *In Vitro*

**DOI:** 10.3389/fphar.2021.650448

**Published:** 2021-05-14

**Authors:** Jiayue Zhu, Xinwei Yang, Xiao Li, Shuo Han, Yanbo Zhu, Liping Xu

**Affiliations:** ^1^ School of Traditional Chinese Medicine, Capital Medical University, Beijing, China; ^2^ Beijing Key Lab of TCM Collateral Diasease Theory Research, Capital Medical University, Beijing, China

**Keywords:** diabetic peripheral neuropathy, Schwann cells, mitochondrial dynamics, Mfn1, Mfn2, OPA1, Drp1

## Abstract

Tang Luo Ning (TLN), a traditional Chinese compound prescription, has been used clinically to treat diabetic peripheral neuropathy (DPN) in China. However, the exact mechanisms remain unclear. The objective of this study is to unravel the effects of TLN on mitochondrial dynamics of DPN in streptozotocin-induced rat models and Schwann cells cultured in 150 mM glucose. Mitochondrial function was determined by Ca^2+^ and ATP levels of streptozotocin (STZ)-induced DPN rats and mitochondria structure, mitochondrial membrane potential (MMP), and mtDNA of high glucose incubated SCs. Mitochondrial dynamics protein including mitofusin 1 (Mfn1), mitofusin 2 (Mfn2), optic atrophy 1 (Opa1), and dynamin-related protein 1 (Drp1) were investigated using Western blot or immunofluorescence. Myelin basic protein (MBP), myelin protein zero (MPZ), and sex-determining region Y (SRY)-box 10 (Sox10) were measured to represent schwannopathy. Our results showed that TLN increased ATP levels (0.38 of model, 0.69 of HTLN, 0.61 of LTLN, *P*＜0.01; 0.52 of 150 mM glucose, 1.00 of 10% TLN, *P*＜0.01, 0.94 of 1% TLN, *P*＜0.05), MMP (0.56 of 150 mM glucose, *P*＜0.01, 0.75 of 10% TLN, *P*＜0.05, 0.83 of 1% TLN, *P*＜0.01), and mtDNA (0.32 of 150 mM glucose, 0.43 of 10% TLN, *P*＜0.01) while decreased Ca^2+^ (1.54 of model, 1.06 of HTLN, 0.96 of LTLN, *P*＜0.01) to improve mitochondrial function *in vivo* and *in vitro*. TLN helps maintain balance of mitochondrial dynamics: it reduces the mitochondria number (1.60 of 150 mM glucose, 1.10 of 10% TLN, *P*＜0.01) and increases the mitochondria coverage (0.51 of 150 mM glucose, 0.80 of 10% TLN, 0.87 of 1% TLN, *P*＜0.01), mitochondrial network size (0.51 of 150 mM glucose, 0.95 of 10% TLN, 0.94 of 1% TLN, *P*＜0.01), and branch length (0.63 of 150 mM glucose, *P*＜0.01, 0.73 of 10% TLN, *P*＜0.05, 0.78 of 1% TLN, *P*＜0.01). Further, mitochondrial dynamics–related Mfn1 (0.47 of model, 0.82 of HTLN, 0.77 of LTLN, *P*＜0.01; 0.42 of 150 mM glucose, 0.56 of 10% TLN, 0.57 of 1% TLN, *P*＜0.01), Mfn2 (0.40 of model, 0.84 of HTLN, 0.63 of LTLN, *P*＜0.01; 0.46 of 150 mM glucose, 1.40 of 10% TLN, 1.40 of 1% TLN, *P*＜0.01), and Opa1 (0.58 of model, 0.71 of HTLN, 0.90 of LTLN, *P*＜0.01; 0.69 of 150 mM glucose, 0.96 of 10% TLN, 0.98 of 1% TLN, *P*＜0.05) were increased, while Drp1 (1.39 of model, 0.96 of HTLN, 1.18 of LTLN, *P*＜0.01; 1.70 of 150 mM glucose, 1.20 of 10% TLN, 1.10 of 1% TLN, *P*＜0.05), phosphorylated Drp1 (2.61 of model, 1.44 of HTLN, *P*＜0.05; 2.80 of 150 mM glucose, 1.50 of 10% TLN, 1.30 of 1% TLN, *P*＜0.01), and Drp1 located in mitochondria (1.80 of 150 mM glucose, 1.00 of 10% TLN, *P*＜0.05) were decreased after treatment with TLN. Additionally, TLN improved schwannopathy by increasing MBP (0.50 of model, 1.05 of HTLN, 0.94 of HTLN, *P*＜0.01; 0.60 of 150 mM glucose, 0.78 of 10% TLN, *P*＜0.01, 0.72 of 1% TLN, *P*＜0.05), Sox101 (0.41 of model, 0.99 of LTLN, *P*＜0.01; 0.48 of 150 mM glucose, 0.65 of 10% TLN, *P*＜0.05, 0.69 of 1% TLN, *P*＜0.01), and MPZ (0.48 of model, 0.66 of HTLN, 0.55 of HTLN, *P*＜0.01; 0.60 of 150 mM glucose, 0.78 of 10% TLN, *P*＜0.01, 0.75 of 1% TLN, *P*＜0.05) expressions. In conclusion, our study indicated that TLN’s function on DPN may link to the improvement of the mitochondrial dynamics, which provides scientific evidence for the clinical application.

## Introduction

Diabetic peripheral neuropathy (DPN) is one of the most common chronic complications of diabetes, which belongs to neurodegenerative diseases (Goncalves et al., 2018). At present, there are no modifiable treatments for DPN other than glucose control and lifestyle modifications (Pop-Busui et al., 2017; Cheng et al., 2019). A number of molecular mechanisms have been proposed, including the polyol pathway hyperactivity, the accumulation of advanced glycation end products (AGE), diacylglycerol-PKC pathway activation, increased poly (ADP-ribose) polymerase (PARP) activity, oxidative stress, increased inflammation, and a reduction in neurotrophic factors (Brownlee, 2005). But, all clinical trials aimed at altering the progressive course of DPN have failed (Pop-Busui et al., 2017). Therefore, seeking effective treatments to improve DPN is still urgent.

Tang Luo Ning (TLN) is a traditional Chinese medicine, which has been used in preventing and treating DPN patients for many years with great clinical effects (Li et al., 2011; Gao et al., 2013). Our previous study demonstrated that TLN could alleviate the injury of the peripheral nerve by improving the nerve conduction velocity and temperature sensation of DPN rats (Yang et al., 2015). The possible mechanism was related to reduce oxidative stress and ER stress, which exacerbate mitochondrial dysfunction (Yang et al., 2017). TLN regulates the PERK/Nrf2 pathway, which plays an important role in mitochondrial dysfunction and decreases the reactive oxygen species (ROS) in the DPN rats both *in vivo* and *in vitro* (Yang et al., 2015; Yang et al., 2017).

Reactive oxygen species (ROS) is one of the regulators of mitochondrial dynamics (Cid-Castro et al., 2018). Mitochondrial dynamics includes fission and fusion. Mitochondria undergo two different but coordinate fusion events: mitofusin 1 (Mfn1) and mitofusin 2 (Mfn2) regulate the fusion of the outer mitochondrial membrane (OMM), and optic atrophy 1 protein (Opa1) controls fusion of the inner mitochondrial membrane (IMM). For mitochondrial fission to occur, the recruitment of dynamin-related protein 1 (Drp1) from the cytosol to the OMM by several mitochondrial membrane adaptors is necessary (Zhang et al., 2019). Mitochondrial dynamics is the therapeutic strategy to improve the mitochondrial functions and is identified as a key factor in neurodegenerative diseases such as Parkinson's disease (PD), Alzheimer's disease (AD), Huntington's disease (HD), amyotrophic lateral sclerosis (ALS), and diabetes (Panchal and Tiwari, 2019; Rumora et al., 2019). But it is not clear whether mitochondrial dynamics interferes with DPN, and whether TLN has beneficial effects on mitochondrial dynamics is yet to be elucidated.

The major pathological features in DPN include axonal atrophy and demyelination. Earlier studies have shown that TLN can reduce apoptosis induced by mitochondrial damage in a high glucose environment (Yang et al., 2016). Here, we found that TLN increased mitochondrial fusion–related Mfn1, Mfn2, and Opa1 while decreasing mitochondrial fission–related Drp1 located on mitochondria by inhibiting Drp1 expression and phosphorylation, thus markedly improving mitochondrial function of DPN rats. Moreover, TLN diminished Schwann cell dysfunction by increasing MBP, Sox10, and MPZ expressions.

## Materials and Methods

### Preparation of TLN

The formula of TLN per dose is listed in Supplementary Table S1. Identification and qualification of the main chemical constituents in TLN extract have been executed, and the protocol for preparation of TLN powder was as previously described (Yang et al., 2015). Eleven compounds of TLN were characterized using HPLC-MS/MS, and five compounds were quantified. The quality control and stability of TLN were investigated by fingerprint.

### Treatment of Diabetic Rats With TLN

Male Sprague–Dawley (SD) rats (weighing 200 ± 20 g) were obtained from the Experimental Animal Center at Capital Medical University, Beijing, China (SCXK 2012–0001), and were housed on a 12-h on and 12-h off light cycle at 23 ± 2°C in rooms with 55 ± 10% relative humidity and allowed free access to drinking water and standard laboratory chows. The experimental procedures were approved by the Ethics Review Committee for Animal Experimentation of Capital Medical University (Ethical Approval Number AEEI-2015-118). Experimental design, protocols, and sample collections were the same as previously described (Yang et al., 2015) and shown in Supplementary Figure S1.

### Calcium Content in DPN Rats

After removal of the sciatic nerve, blood samples were collected and centrifuged at 3000 rpm for 10 min at 4°C to obtain serum. All serum samples were stored at −80°C. A Calcium Assay Kit (Nanjing Jiancheng Bioengineering Institute, Jiangsu, China) was used to examine serum Ca^2+^ levels. All experimental procedures were performed according to the manufacturer’s instructions.

### TLN Serum Preparation

SD rats were intragastrically administered with distilled water, 20 mg/kg/day ALA suspension, and 10.9 g crude drug/kg/day TLN, respectively, for 7 days. Rats were anesthetized using a small animal anesthesia machine (Matrx VMR, Midmark). Blood was sterilely collected through the ventral aorta. After settling for 2 h at room temperature, the blood samples were centrifuged at 3000 rpm/min at 4°C for 15 min and inactivated at 56°C for 30 min. The samples were stored at −80°C after filtered through a microfiltrate membrane (0.22 μm).

### Treatment of RSC96 Cells Cultured With 150 mM Glucose and TLN

RSC96 cells (CRL-2765) were purchased from the American Type Culture Collection (ATCC) and were cultured in DMEM (No. 11965-092, Gibco) and 10% FBS at 37°C in a humidified atmosphere of 5% CO_2_. Cells were allowed to adhere for 24 h and then treated with 25 mM glucose (containing 10% normal rat serum), 150 mM glucose (containing 10% normal serum), 10% ALA (150 mM glucose +10% ALA serum), 10% TLN (150 mM glucose +10% TLN serum), and 1% TLN (150 mM glucose +1% TLN serum +9% normal serum) for 48 h.

### ATP

For ATP of the sciatic nerve, the left sciatic nerves stored at −80°C were made into 10% homogenate (tissue gram/phosphate saline buffer in millilite = 1:9) and used for ATP measurement using an ATP enzyme test kit (Nanjing Jiancheng Bioengineering Institute) according to the manufacturer’s guidelines. A BCA protein kit was used to measure the protein content in samples according to the manual. The ATP level in cells was determined using the Luminescent ATP Detection Assay Kit (ab113849, Abcam) following the manufacturer’s protocol.

### Mitochondrial Structure

Cells were plated in 100-mm confocal dish and washed with HBSS and fixed using Gluta fixative (2.5% Glutaraldehyde, EM Grade, P1126, Solarbio). Images were acquired using a Hitachi HT7700 Transmission Electron Microscope. The mitochondria number and mitochondria coverage were quantified using ImageJ software (Dietrich et al., 2013). In addition, mitochondria was measured by incubating with MitoTracker Green FM (200 nM, M7154, Thermo Fisher) for 30 min at 37°C in the dark according to the manufacturer’s instructions (Ghosh et al., 2019). After incubation, the cells were washed twice with pre-warmed DMEM. Fluorescent images were captured by a Zeiss LSM 880 with Airyscan. Images were analyzed by using Fiji software.

### Mitochondrial Membrane Potential

Determination of ΔΨm was performed using TMRM (Ghosh et al., 2019). Cells were cultured in a 20-mm confocal dish and incubated with Image-iT™ TMRM Reagent (200 nM, I34361, Thermo Fisher) and MitoTracker™ Red FM (200 nM, M22425, Thermo Fisher) for 30 min at 37°C according to the manufacturer’s instructions. After incubation, the cells were washed twice with pre-warmed DMEM. Fluorescent images were captured by a Zeiss LSM 880 with Airyscan. Images were analyzed using Image-Pro Plus 6.0 software.

### Mitochondrial DNA (mtDNA)

mtDNA was detected by expression of the mtDNA-binding protein TFAM (transcription factor A, mitochondrial) (Mcarthur et al., 2018). Cells plated in a 20-mm confocal dish and incubated with MitoTracker TM Deep Red FM (200 nM, M22426, Thermo Fisher), followed by fixing with 4% paraformaldehyde for 10 min. 0.1% Triton X-100 for 10 min and 3% bovine serum albumin (BSA)/PBS for 30 min were used to permeabilize and block the nonspecific binding sites. Then, cells were incubated with the mtTFA antibody (1:500, ab176558, Abcam) and donkey anti-rabbit IgG-H&L (Alexa Fluor 488) in 1.5% BSA. Fluorescent images were captured by a Zeiss LSM 880 with Airyscan. Images were analyzed using Image-Pro Plus 6.0 software.

### Immunostaining

A segment of sciatic nerves (1 cm) was fixed in 10% buffered formalin; 5-µm-thick transverse sections were repaired using antigen retrieval with hot citric acid buffer (pH 6.0) and blocked with 3% BSA. The sections were incubated with primary antibodies (Mfn1, 1:200, OM209507, omnimabs; Opa1, 1:200, ab157457, Abcam; Drp1, 1:200, ab184247, Abcam; MPZ, 1:50, ab183868, Abcam; S100, 1:100, ab4066, Abcam) at 4°C overnight followed by incubation with the secondary antibody and incubated at room temperature for 2 h. The sections were observed using Leica TCS SP8 STED and a Leica LAS image acquisition system. Image-Pro Plus 6.0 software was used for image analysis. Cells were plated in a 20-mm confocal dish and incubated with MitoTracker TM Deep Red FM and Drp1 antibody (1:250, ab184247, Abcam), as described in methods of 2.9.

### High-Content Assay

Cells were plated in a 96-well plate and fixed, permeabilized, and blocked, as described in 2.9. Then, the cells were incubated with Oma1 (1:200, ab154949, Abcam) at 4°C for 18 h, respectively. And the cells were incubated with secondary antibodies at room temperature for 1 h. DAPI was used to stain the nucleus. Automated cell image acquisition was performed on a Thermo Fisher Scientific Cellomics ArrayScan VTI High-Content Screening Reader. Image analysis was done using the assorted Compartmental Analysis Bioapplication (Yang et al., 2016).

### Western Blot

Proteins from sciatic nerves or cells were separated using RIPA and centrifuged at 12,000 g for 15 min at 4°C. Proteins were denatured at 95°C for 5 min and analyzed on SDS-PAGE, transfer to the PVDF membrane, and immunoblotting for Mfn2 (1:1000, ab124773, Abcam), MPB (1:1000, ab62631, Abcam), Sox10 (1:1000, ab155279, Abcam), Phospho-Drp1(Ser616) (1:1000, 4494, CST), OPA1 (1: 1000, ab157457, Abcam), Mfn1 (1:500, OM209507, omnimabs), Drp1 (1:1000, ab184247, Abcam), and β-actin (1:2000, TA-09, ZSGB) was performed overnight at 4°C.

### Statistical Analysis

Data presentation and statistical analyses were carried out using Prism 7.04 software (Graph Pad) and graphed as mean ± standard error of the mean (S.E.M). Differences were analyzed by one-way ANOVA followed by Tukey’s multiple comparisons test. Student’s unpaired t test was used to analyze data between 2 groups. P < 0.05 was considered to be statistically significant.

## Results

### Effects of TLN on Mitochondrial Function in DPN Rats

It has been reported that DPN is usually accompanied with demyelination of sciatic nerves. Our previous studies have confirmed that TLN treatment improved diabetes-induced demyelination in sciatic nerves and was consistent with the function of sciatic nerves (Yang et al., 2015; Yang et al., 2017). For this study, we confirmed the protective effect of TLN on the structure and function of sciatic nerves again; results are given in Supplementary Figure S2.

**FIGURE 1:**
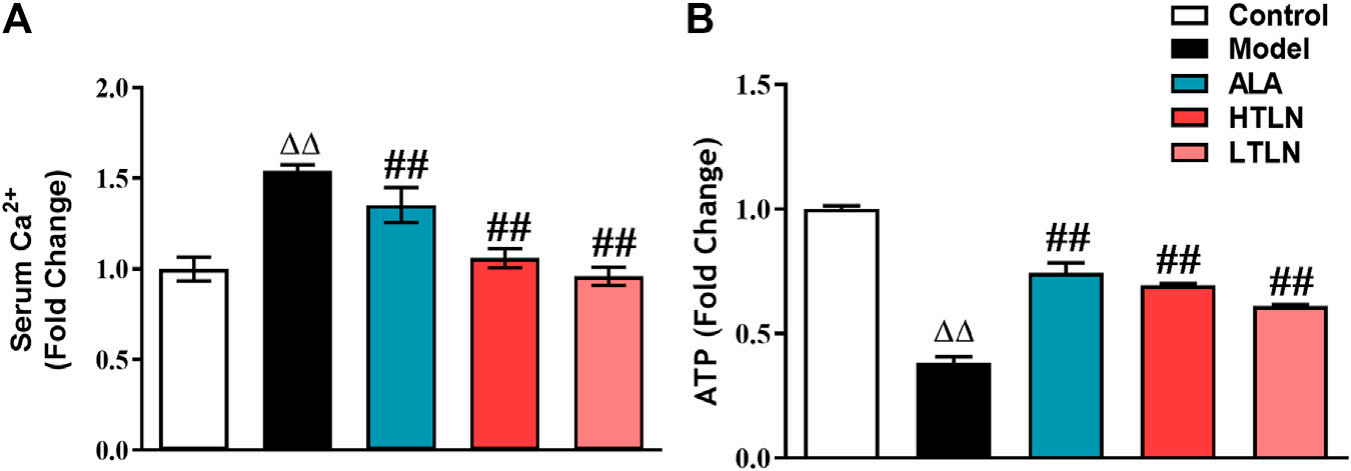
TLN treatment on mitochondrial function in DPN rats **(A)** Serum Ca^2+^ levels of rats were measured. **(B)** ATP levels in sciatic nerves were measured. n = 6 for each group. ^ΔΔ^ *P*< 0.01 vs*.* control; ^##^ *P* < 0.01 vs*.* model.

To investigate whether TLN has effects on mitochondrial function in DPN, we detected sciatic nerve ATP levels and Ca^2+^ in rat serum. As shown in Figure 1A, compared with the control group, the serum Ca^2+^ level in the model group was significantly increased (*P* < 0.01), while TLN decreased the Ca^2+^ level in DPN rats (*P* < 0.01). Meanwhile, TLN increased the ATP content in sciatic nerves (*P* < 0.01) (Figure 1B). These data demonstrate that TLN improves mitochondrial function in DPN rats.

### Effects of TLN on Mitochondrial Dynamics in DPN Rats

**FIGURE 2:**
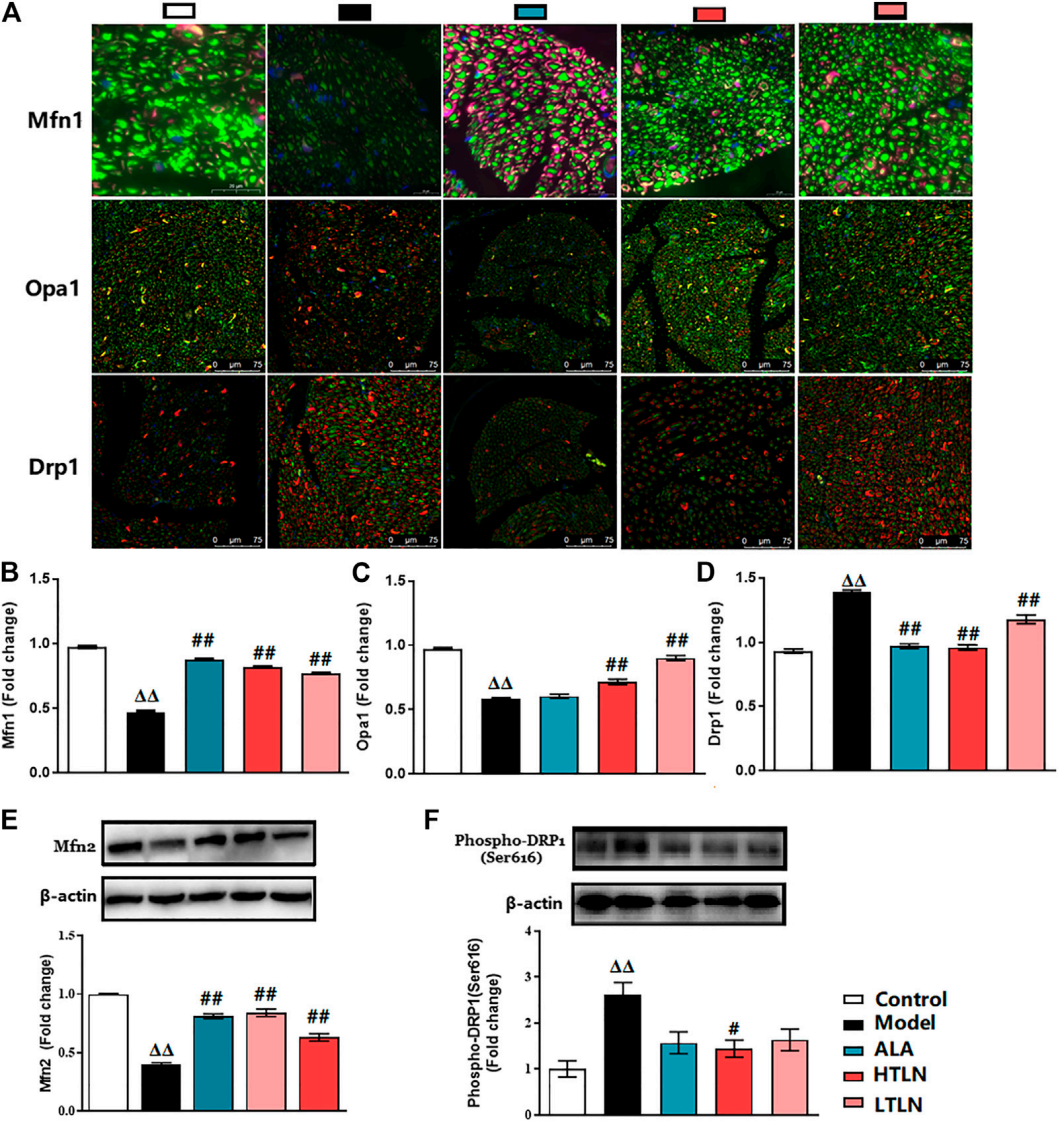
TLN treatment on mitochondrial dynamics in DPN rats. **(A)** Representative images of immunofluorescence staining on Mfn1 (green); scale bar, 20 μm. Opa1 (green); scale bar, 75 μm. Drp1 (green); scale bar, 75 μm. Schwann cells are stained with S100β (red). **(B-D)** Quantifications of indicated proteins. Data were presented as fold change of the control group. **(E, F)** Western blotting of Mfn2 and phospho-Drp1(Ser616) of sciatic nerves and quantifications of this proteins. n = 6 for each group. ^ΔΔ^ *P*< 0.01 vs*.* control; ^**#**^ *P*< 0.05, ^##^ *P* < 0.01 vs*.* model.

To further confirm that TLN has protective effects on DPN, we investigated whether TLN can improve DPN-induced imbalance of mitochondrial dynamics. As expected, TLN increases the expression of mitochondrial fusion proteins; meanwhile, TLN decreases the expression of mitochondrial fission protein in Schwann cells of sciatic nerves in DPN rats. Compared with the model group, Mfn1/2 and OPA1 expression levels in sciatic nerves were increased significantly in the TLN group (*P* < 0.01, Figures 2A–C,E). Compared with the model group, Drp1 and phospho-Drp1(Ser616) levels in sciatic nerves were decreased significantly in the TLN group (*P* < 0.05 or *P* < 0.01, Figure 2D,F). These data demonstrate that TLN can regulate DPN-induced imbalance of mitochondrial dynamics and further confirmed the protective effects of TLN on DPN.

### Effects of TLN on Mitochondrial Structure and Function of SCs

**FIGURE 3:**
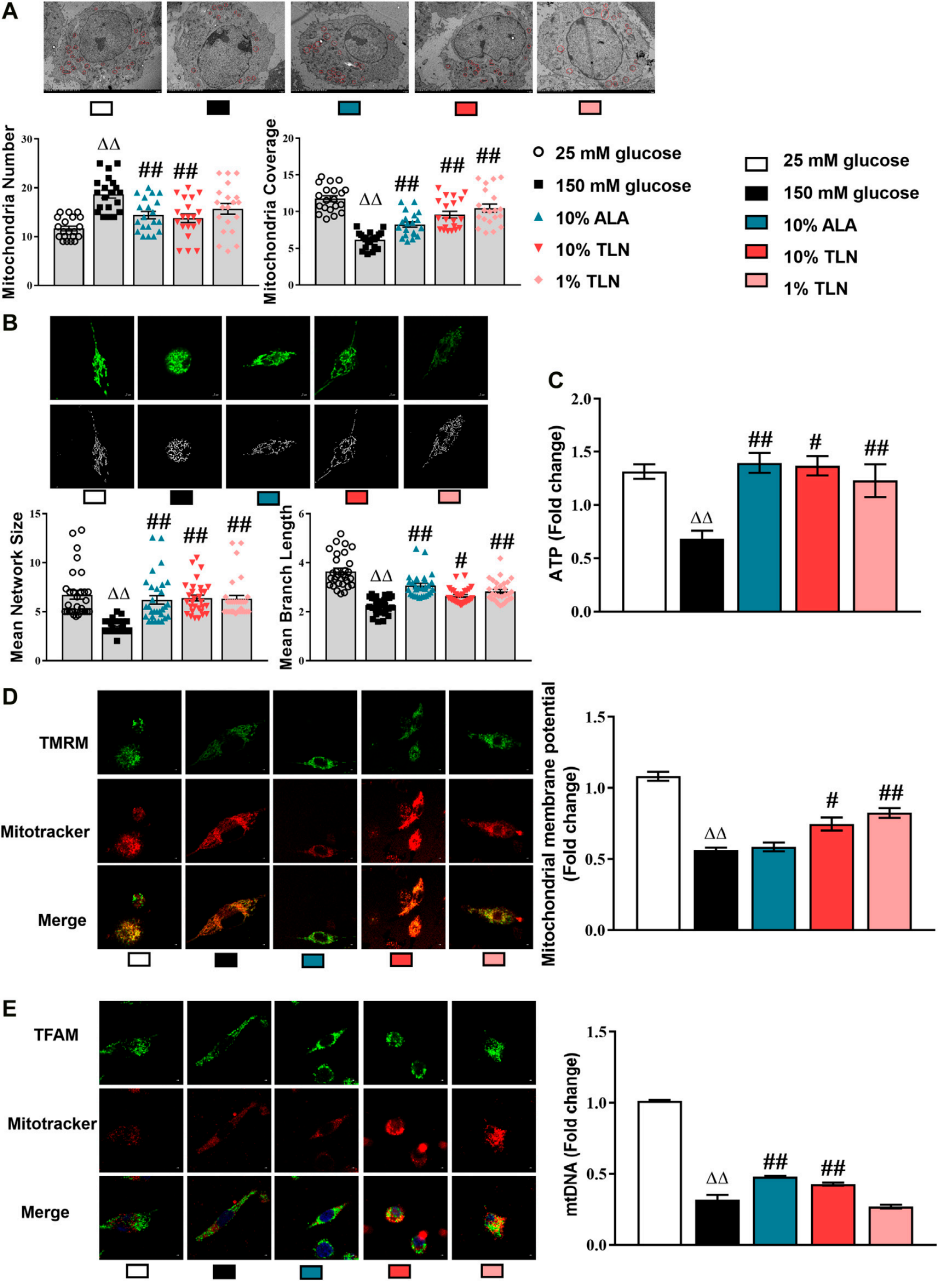
TLN serum treatment improved the mitochondrial structure and function of SCs incubated in a high glucose environment. **(A)** Representative images and quantifications of the mitochondria number and mitochondria coverage of 30 cells. Scale bar, 5 μm. The results were normalized to the values of the 25 mM glucose group. Mitochondria are indicated by red circles. **(B)** Representative images and quantifications of the mean network size and mean branch length of 20 cells. Scale bar, 5 μm. **(C)** Quantification of ATP, n = 4 for each group. **(D)** Representative images of immunofluorescence staining on TMRM (green) and mitochondria (red); scale bar, 5 μm, and quantification of mitochondrial membrane potential, n = 4 for each group. **(E)** Representative images of immunofluorescence staining on TFAM (green) and mitochondria (red); scale bar, 5 μm, and quantification of mtDNA, n = 4 for each group. ^ΔΔ^ *P*< 0.01 vs*.* 25 mM glucose group; ^##^ *P* < 0.01, ^#^ *P*< 0.05 vs. 150 mM glucose group.

To further verify the mechanism of TLN intervention on DPN, we studied SCs in a high glucose environment. First, we observed its effect on the mitochondrial structure. After 48 h of high glucose treatment, the mitochondria coverage, mitochondria network size, and mitochondria branch length were significantly decreased and the mitochondria number was significantly increased in the 150 mM glucose group compared with the 25 mM glucose group, whereas TLN treatment significantly increased the mitochondria coverage, mitochondria network size, and mitochondria branch length and decreased the mitochondria number compared to the 150 mM glucose group **(** *P*< 0.01 or *P*< 0.05, Figures 3A,B). It is shown that TLN may increase the SC mitochondrial fusion and decrease fission in the high glucose environment. Likewise, mtDNA was significantly lower in the 150 mM glucose group than the 25 mM glucose group, and TLN groups showed a higher mtDNA than the 150 mM glucose group **(** *P* < 0.01, Figure 3E **).**

ATP was significantly decreased in the 150 mM glucose group compared with the 25 mM glucose group, whereas TLN-treated SCs displayed a lower ATP than the high-glucose–incubated SCs (*P*< 0.01 or *P*< 0.05, Figure 3C **).** Additionally, the levels of mitochondrial membrane potential (MMP) were lower in the 150 mM glucose group than the 25 mM glucose group and were higher in the TLN treatment groups than the 150 mM glucose group **(** *P*< 0.01 or *P*< 0.05, Figure 3D **).** Collectively, these data confirm that TLN treatment improves the mitochondrial structure and function, likely affecting multiple steps of mitochondrial dynamics.

### Effects of TLN on Mitochondrial Dynamics of SCs

**FIGURE 4:**
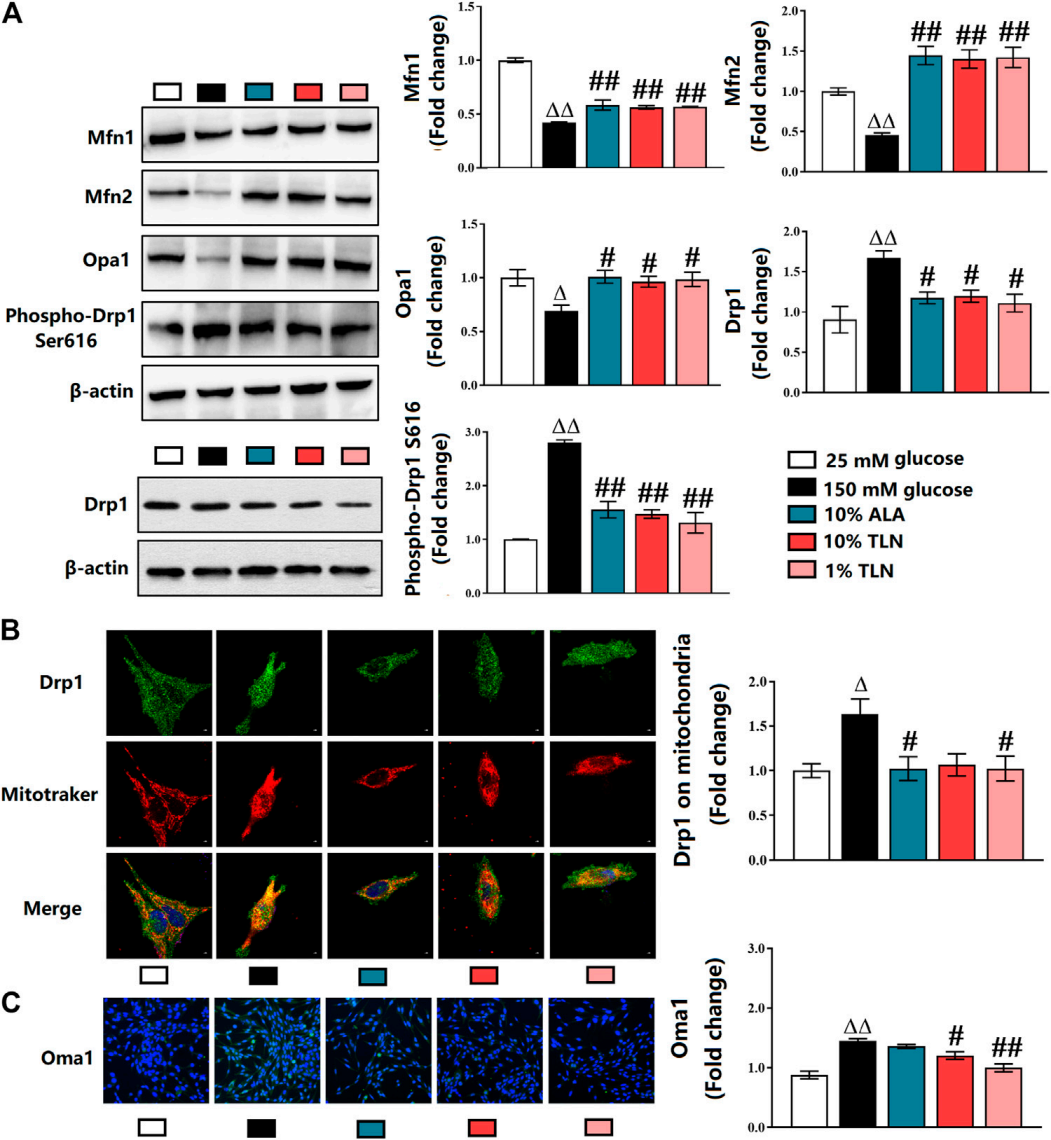
TLN serum treatment increased mitochondrial fusion, while decreased fission of SCs incubated in a high glucose environment. **(A)**Western blotting of Mfn1, Mfn2, Opa1, Drp1, and phospho-Drp1 (Ser616) of SCs and quantifications of these proteins. The results were normalized to the values of the 25 mM glucose group. β-actin images are reused. **(B)** Representative images of immunofluorescence staining on Drp1 (green), mitochondria (red), and nucleus (blue). Scale bar, 5 μm, and quantification of Drp1 located on mitochondria, n = 4 for each group. **(C)** Representative images of immunofluorescence staining on Oma1 (green) and nucleus (blue), magnification 40×, and quantification of Oma, n = 4 for each group. ^ΔΔ^ *P*< 0.01, ^Δ^ *P*< 0.05 vs*.* 25 mM glucose group; ^##^ *P* < 0.01, ^#^ *P*< 0.05 vs*.* 150 mM glucose group.

We, therefore, dissect the mitochondrial dynamics–related proteins in the SCs. Our results showed significantly lower activities of Mfn1, Mfn2, and OPA1 and higher levels of Drp1 and phosphorylated Drp1 in the 150 mM glucose group compared with the 25 mM glucose group. Compared with the 150 mM glucose group, the activities of Mfn1, Mfn2, and OPA1 were higher, and levels of Drp1 and phosphorylated Drp1 were lower (*P*< 0.01 or *P*< 0.05, Figure 4A). As shown in Figure 4B, Drp1 located on mitochondria was decreased in SCs incubated with 150 mM glucose when compared to cells with 25 mM glucose, and TLN treatment increased Drp1 located on mitochondria (*P*< 0.05). As indicated in Figure 4C, there was a higher activity of Oma1 in the 150 mM glucose group than the 25 mM glucose group, and TLN treatment made the activity lower.

### Effects of TLN on Schwann Cells Function in DPN Rats

**FIGURE 5:**
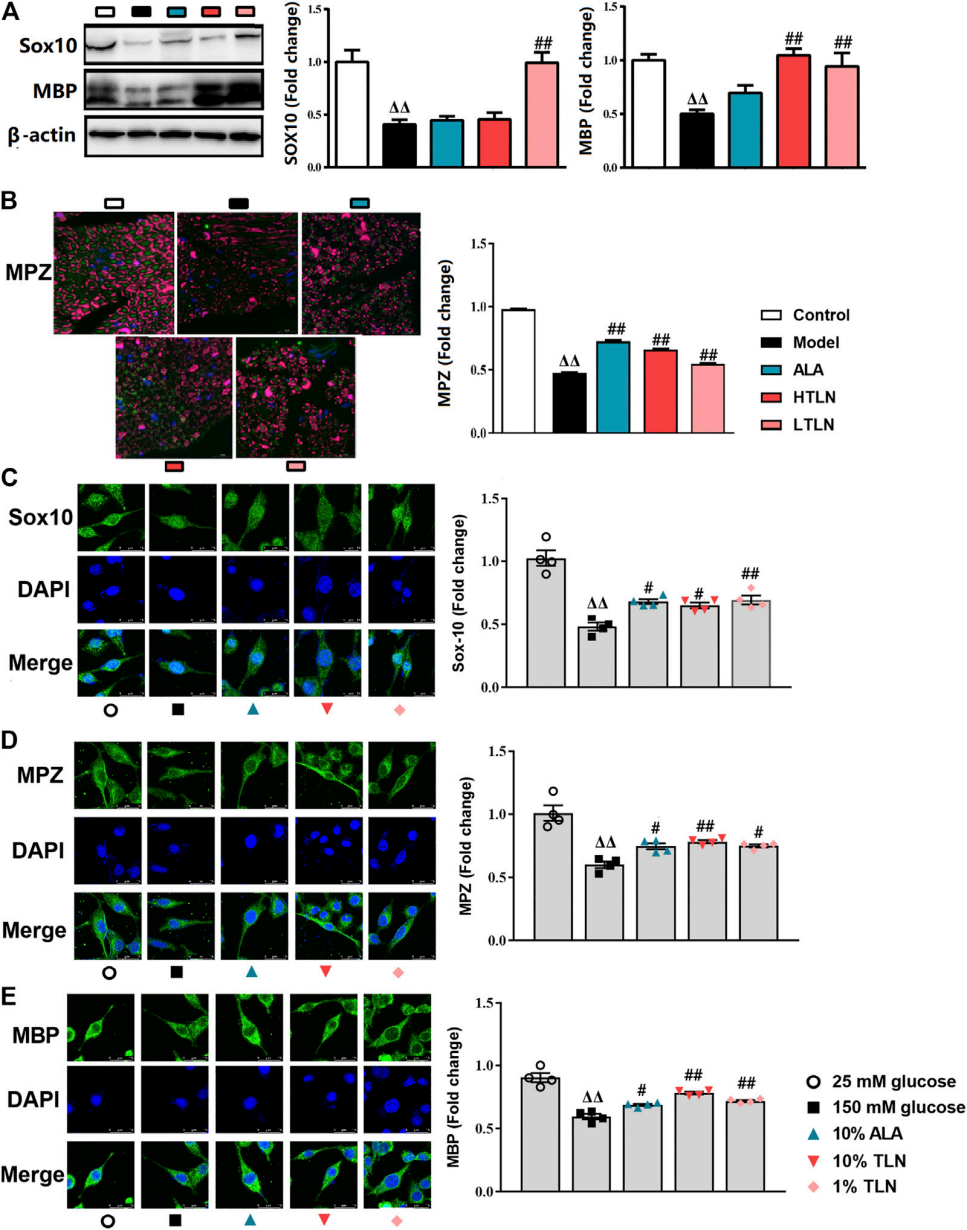
TLN increased MBP, MPZ, and Sox10 *in vivo* and *in vitro* to ameliorate schwannopathy. **(A)**Western blotting of Sox10 and MBP in sciatic nerves of DPN rats and quantifications of these proteins. The results were normalized to the values of the control group, n = 6 for each group. β-actin images are reused. **(B)** Representative images of immunofluorescence staining on MPZ (green) and S100β (purple). Scale bar, 20 μm, and quantification of MPZ, n = 6 for each group. ^ΔΔ^ *P*< 0.01, ^Δ^ *P*< 0.05 vs*.* control group; ^##^ *P* < 0.01, #*P*< 0.05 vs. model group **(C–E)** Representative images of immunofluorescence staining on Sox10 (green), MPZ (green), MBP (green), and nucleus (blue). Scale bar, 5 μm, and quantification of Sox10, MPZ, and MBP, n = 4 for each group. ^ΔΔ^ *P*< 0.01, ^Δ^ *P*< 0.05 vs*.* 25 mM glucose group; ^##^ *P* < 0.01, ^#^ *P*< 0.05 vs*.* 150 mM glucose group.

We speculate that TLN can increase the key proteins essential for myelination by regulating mitochondrial dynamics. To confirm that, we measured myelin basic protein (MBP), myelin protein zero (MPZ), and sex-determining region Y (SRY)-box 10 (Sox10) *in vivo* and *in vitro*. We found that the level of MBP, MPZ, and Sox10 in sciatic nerves was similarly increased in rats treated with TLN **(** *P* < 0.05 or *P* < 0.01, Figures 5A,B). The results were also validated in TLN serum–treated SCs (Figures 5C–E).

## Discussion

Our previous studies have found that TLN can reduce demyelination of sciatic nerves in DPN rats and improve MNCV and SNCV. Further studies showed that the improvement of the neural structure and function was associated with upregulation of the NF-E2–related factor 2 (Nrf2)/antioxidant responsive element (ARE) endogenous antioxidant stress pathway to reduce oxidative stress as well as inhibiting the bcl-2–associated X protein (Bax), cytochrome C (Cyto C), and caspase-3 in the mitochondrial apoptosis pathway (Yang et al., 2015; Yang et al., 2017). Comprehensive analysis showed that the improvement of the structure and function of the sciatic nerve in DPN rats was related to the influence of factors related to mitochondrial function to reduce Schwann cell (SC) lesions (Yang et al., 2016). In addition, the highest five contents in TLN extract are salvianolic acid B, paeoniflorin, albiflorin, rosmarinic acid, and chlorogenic, which represent the material basis for the observed neuroprotective and mitochondrial function regulation effects.

Mitochondrial reactive oxygen species (ROS) over formation induced by hyperglycemia is considered to be the unifying pathogenesis of diabetes complications including DPN (Brownlee, 2005). Studies have shown that a high glucose environment can cause abnormalities in the modification of vital enzymes and proteins involved in mitochondrial function, such as ROS production, mitochondrial dynamics, and mitochondrial stress (Agarwal et al., 2020), suggesting that a high glucose environment is closely related to mitochondrial function.

Emerging studies have confirmed that imbalance of mitochondrial dynamics which influence mitochondria morphology leading to peripheral neuropathy including Charcot–Marie–Tooth disease, ALS, and diabetes (Rovira-Llopis et al., 2017; Whitley et al., 2019; Rumora et al., 2019; Zhang et al., 2019). It is strongly suggested that mitochondrial dynamics and dysfunction may be involved in SCs lesions, but how to interfere with DPN by SCs mitochondrial dynamics has not been clarified, which is a problem proposed and urgently needed to be solved. The processes of mitochondrial fission and fusion are regulated by the large GTPases. Mitofusin 1 and mitofusin 2 control fusion of the OMM, whereas Opa1 coordinates IMM. Drp1, through association with specific mitochondria-localized receptors, drives fusion of both IMM and OMM (Simula et al., 2019). Hyperglycemia has been reported to impair mitochondrial function and dynamics associated with the development of neuropathy. But how mitochondrial dynamics involves in DPN is unclear.

In this study, we observed that Mfn1, Mfn2, and OPA1 decrease in sciatic nerves of DPN rats, and TLN can increase these expressions. Downregulation of Mfn2 has been reported to lead to the impairment of mitochondrial fusion and calcium homeostasis, as well as increase the Bax level in mitochondria, causing neuronal death (Martorell-Riera et al., 2014). A recent study demonstrated that overexpression of peroxisome proliferator–activated receptor-gamma co-activator 1α (PGC-1α) can stimulate Mfn2 expression and increase mitochondrial fusion activity (Chandhok et al., 2018). PGC-1α activates transcriptional factors such as Nrf2 and regulates mitochondrial degeneration in DPN (Choi et al., 2014). Nrf2 is known as the nodal point in the coordination of mitochondrial dynamics (O'Mealey et al., 2016; Rambold and Pearce, 2018; Webb et al., 2019). Our pervious study has demonstrated that TLN can activate Nrf2 and decrease the Bax level (Yang et al., 2015; Yang et al., 2017). This result is in accordance with upregulating expression of Mfn2.

Opa1 is associated with these functions such as mediating mitochondrial dynamics mtDNA maintenance, cristae integrity, cellular redox homeostasis, and apoptosis. TLN increases the Opa1 expression, thus improving cellular redox homeostasis and apoptosis, as previously reported. Excessive fission is associated with increased ROS production (Webb et al., 2019). Oxidative stress will make the mitochondrial proteases Oma1 hyperactivated and then the L-Opa1 form is cleaved to S-Opa1. Accumulation of S-Opa1 has been clarified to cause mitochondrial fission (Wai and Langer, 2016). In this research, high glucose induced oxidative stress and excessive activated Oma1, thus increasing the mitochondrial fission.

It is found that phosphorylation of Drp1 at Ser616 causes increased Drp1-mediated mitochondrial fission, leading to cell death under oxidative stress (Zhang et al., 2019). Posttranslational modifications of Drp1 include SUMOylation. SUMOylation of Drp1 promotes mitochondrial division by stabilizing Drp1 on the mitochondria (Whitley et al., 2019). We further examined the Drp1 located in mitochondria. Comprehensive analysis shows that TLN decreases not only the Drp1 expression but also posttranslational modifications to reduce mitochondrial fission.

Mitochondrial dysfunctions have been observed as early features of several neurodegenerative diseases (Yu and Pekkurnaz, 2018). Mitochondria are the core organelles in cells, which play important roles in maintaining ATP bioenergetics, Ca^2+^ homeostasis, oxidative stress balance, and apoptosis during cell survival (Panchal and Tiwari, 2019). Impaired mitochondrial functions including decreased ATP production and increased Ca^2+^ has been linked with alterations in mitochondrial fission and fusion processes (Bhatti et al., 2016; Panchal and Tiwari, 2019). Mitochondrial fusion events did not affect mitochondrial bioenergetic function, mitochondrial membrane potential (MMP), and ATP production and decrease ROS generation and apoptosis. On the contrary, excessive mitochondrial fission leads to mitochondrial depolarization, impairs mitochondrial function, and reduces ATP and ROS production, leading to apoptosis. Our current experimental results confirm that TLN can increase ATP production as well as restore of Ca^2+^ homeostasis. The previous research has shown that TLN can decrease ROS generation and increase the Schwann cell MMP under high glucose (Yang et al., 2015; Yang et al., 2017). The aberrant mitochondrial structure and mtDNA have been hypothesized to contribute to altered expression of the mitochondrial fusion and division proteins (Whitley et al., 2019). Mitochondrial defects are closely related to peripheral neuropathy. Studies have shown that mtDNA deletion in SCs not only affects the expression of key components of the mitochondrial electron transport chain, resulting in mitochondrial dysfunction and abnormal mitochondrial morphology, but also leads to lipid synthesis disorders, which, in turn, affects peripheral nerve myelination (Paolo and Przedborski, 2013). Our results confirmed that TLN can increase the content of SCs mtDNA in a high glucose environment. This is consistent with the results that TLN increased myelin basic protein (MBP), myelin protein zero (MPZ), and sex determining region Y (SRY)-box 10 (Sox10) to improve demyelination. These observations provide that TLN can improve mitochondrial dynamics and dysfunction to treat DPN.

Schwann cells (SCs) are the most abundant glial cells of the PNS, ensheathing axons of peripheral nerves as myelinating cells. Accumulating data from research have identified schwannopathy as a central mechanism that leads to DPN (Goncalves et al., 2018). Schwannopathy is defined as inhibition of proteins involved in myelin formation including MBP, MPZ, and Sox 10, which results in oxidative damage and apoptosis (Goncalves et al., 2017). TLN regulates mitochondrial dynamics of SCs, thus reducing demyelination of sciatic nerves. This is related to increased expression of MBP, MPZ, and Sox10.

## Conclusion

We found that TLN increased mitochondrial fusion–related Mfn1, Mfn2, and Opa1 while decreasing mitochondrial fission–related Drp1 and phosphorylation^S616^ of Drp1 and its location on mitochondria in Schwann cells of sciatic nerves. This is consistent with the effect of TLN on the structure of mitochondria. In addition, TLN can decrease Ca^2+^ and increase ATP activity in DPN rats, suggesting that TLN can improve mitochondrial function. Based on this, TLN diminished Schwann cell–related demyelination by increasing MBP, Sox10, and MPZ expressions. Taken together, TLN can correct the imbalance of mitochondrial dynamics to improve the function of mitochondria and then ameliorate schwannopathy to reduce demyelination, thus interfering with DPN.

## Data Availability

The raw data supporting the conclusions of this article will be made available by the authors, without undue reservation.
