# Corrigendum: Tang Luo Ning, a Traditional Chinese Compound Prescription, Ameliorates Schwannopathy of Diabetic Peripheral Neuropathy Rats by Regulating Mitochondrial Dynamics In Vivo and In Vitro

**DOI:** 10.3389/fphar.2021.718452

**Published:** 2021-07-22

**Authors:** Jiayue Zhu, Xinwei Yang, Xiao Li, Shuo Han, Yanbo Zhu, Liping Xu

**Affiliations:** ^1^ School of Traditional Chinese Medicine, Capital Medical University, Beijing, China; ^2^ Beijing Key Lab of TCM Collateral DiaseaseTheory Research, Capital Medical University, Beijing, China

**Keywords:** diabetic peripheral neuropathy, schwann cells, mitochondrial dynamics, mfn1, MFN2, OPA1, DRP1

In the original article, there was a mistake in Figure 3 as published. There is a repeated image, but are labeled as different treatments. The corrected Figure 3 appears below.

**FIGURE 3:**
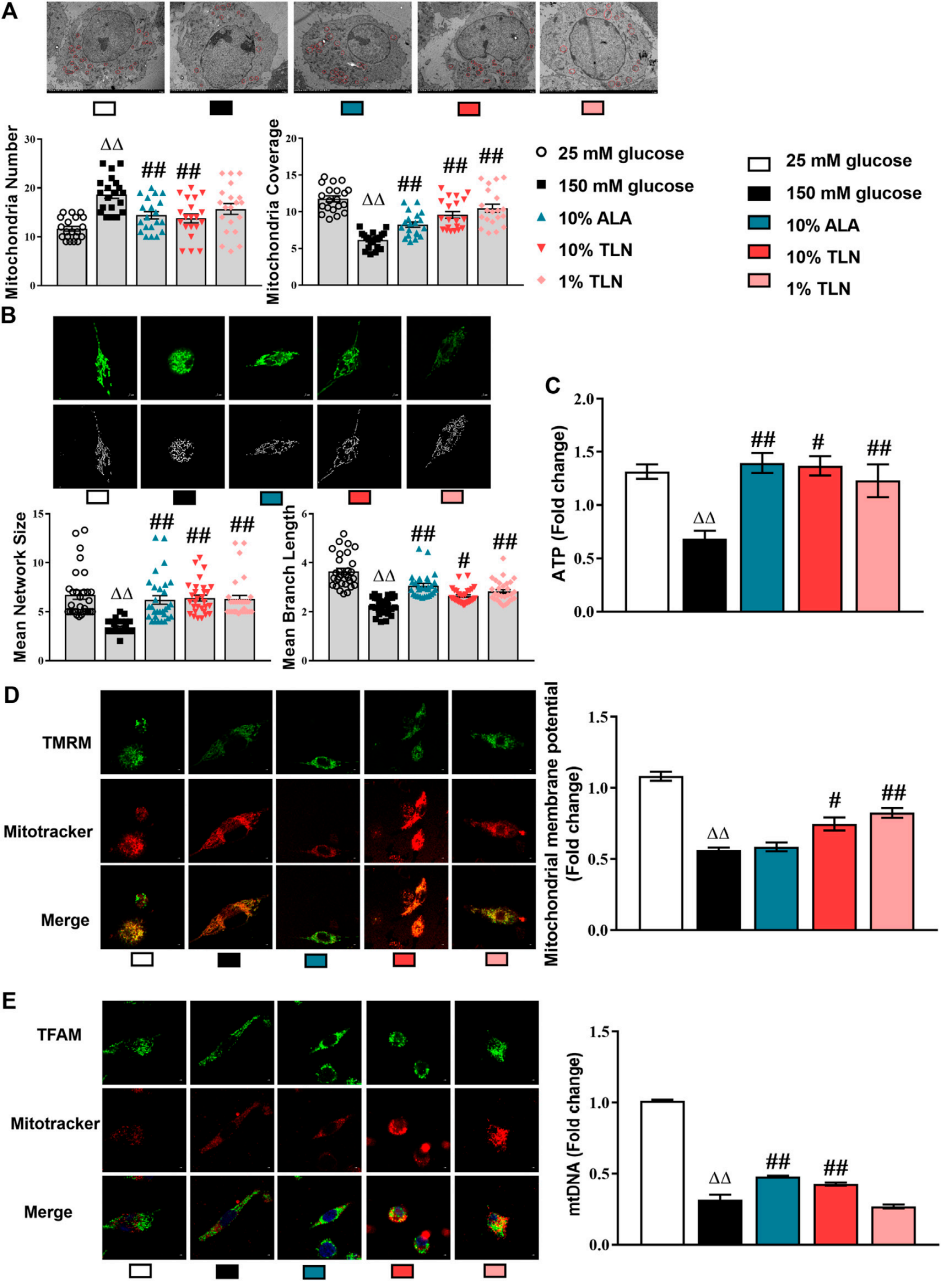
TLN serum treatment improved the mitochondrial structure and function of SCs incubated in a high glucose environment. **(A)** Representative images and quantifications of themitochondria number and mitochondria coverage of 30 cells. Scale bar, 5 μm. The results were normalized to the values of the 25 mM glucose group. Mitochondria are indicated by red circles. **(B)** Representative images and quantifications of themean network size and mean branch length of 20 cells. Scale bar, 5 μm. **(C)** Quantification of ATP, n = 4 for each group. **(D)** Representative images of immunofluorescence staining on TMRM (green) and mitochondria (red); scale bar, 5 μm, and quantification of mitochondrial membrane potential, n = 4 for each group. **(E)** Representative images of immunofluorescence staining on TFAM (green) and mitochondria (red); scale bar, 5 μm, and quantification of mtDNA, n = 4 for each group. ^ΔΔ^ *P*< 0.01 vs. 25 mM glucose group; ^##^ *P* < 0.01, ^#^ *P*< 0.05 vs. 150 mM glucose group.

The authors apologize for this error and state that this does not change the scientific conclusions of the article in any way. The original article has been updated.

