# Supplementary material for: Tang Luo Ning, a Traditional Chinese Compound Prescription, Ameliorates Schwannopathy of Diabetic Peripheral Neuropathy Rats by Regulating Mitochondrial Dynamics In Vivo and In Vitro
Source: Front Pharmacol. 2021 May 14;12:650448. doi: 10.3389/fphar.2021.650448 (PMC8160508; doi:10.3389/fphar.2021.650448)
Supplement: Supplementary file 1 [file Table1.docx]

**Suppl. Table 1 Eight crude drug materials of TLN**

| **Crude drug** | **Dose (g)** |
| --- | --- |
| *Astragalus mongholicus*Bunge | 15 |
| *Salvia miltiorrhiza*Bunge | 15 |
| *Spatholobus suberectus* Dunn | 15 |
| *Chaenomeles lagenaria (Loisel.)* Koidz | 15 |
| *Cibotium Barometz* (L.) J. Sm. | 15 |
| *Achyranthes bidentata*Blume | 12 |
| *Paeonia lactiflflora* Pall*.* | 12 |
| *Corydalis yanhusuo*(Y.H.Chou & Chun C.Hsu) W.T.Wang ex Z.Y.Su & C.Y.Wu | 10 |
