# Supplementary figures and images for: Tang Luo Ning, a Traditional Chinese Compound Prescription, Ameliorates Schwannopathy of Diabetic Peripheral Neuropathy Rats by Regulating Mitochondrial Dynamics In Vivo and In Vitro
Source: Front Pharmacol. 2021 May 14;12:650448. doi: 10.3389/fphar.2021.650448 (PMC8160508; doi:10.3389/fphar.2021.650448)

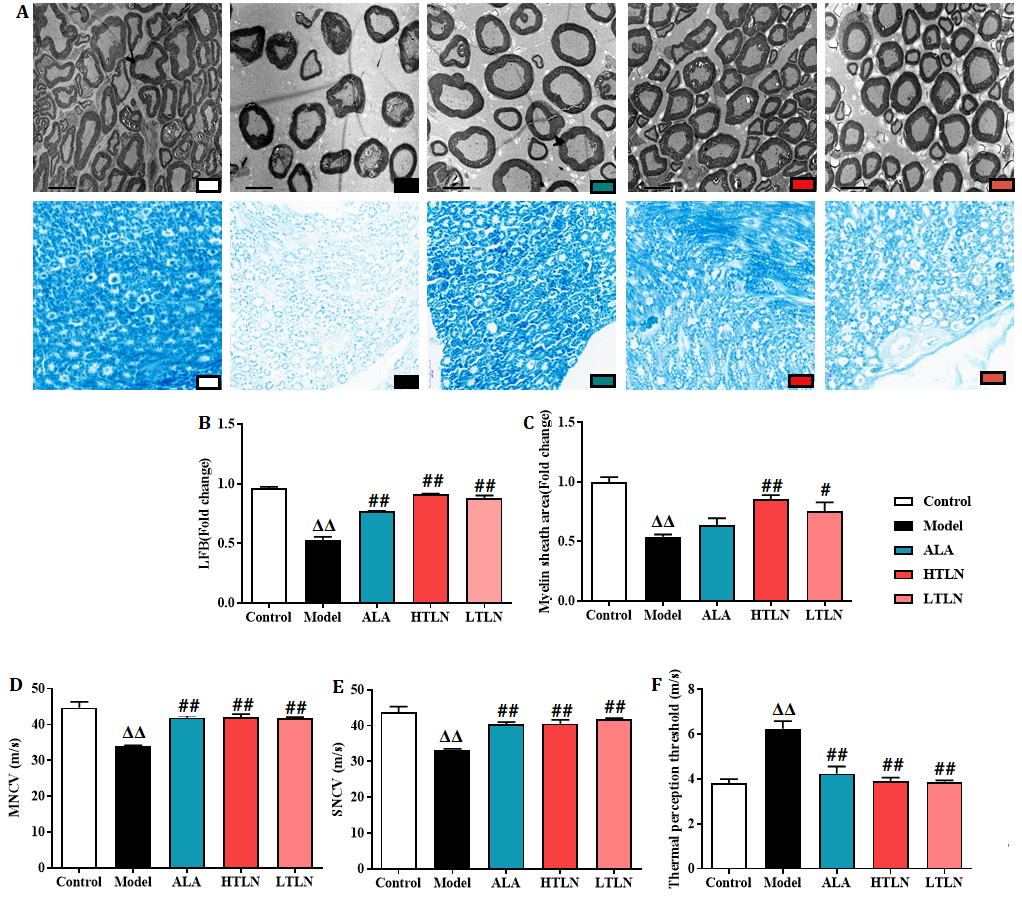

Supplement: Supplementary file 2 [file Image2.TIF]

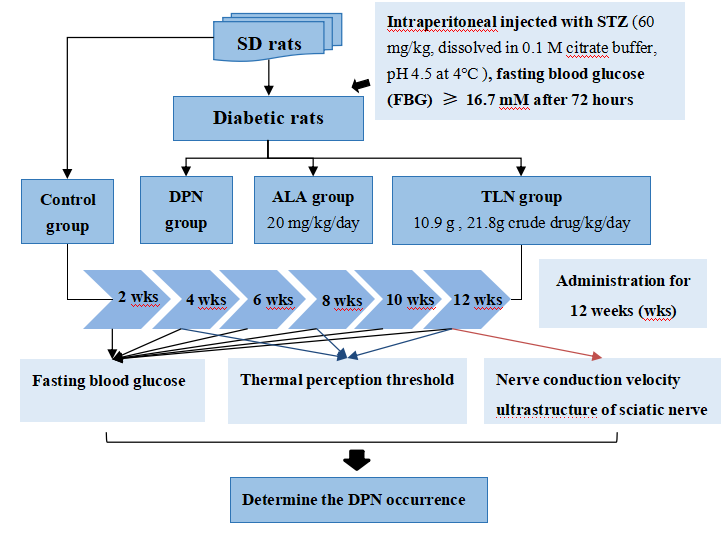

Supplement: Supplementary file 3 [file Image1.TIF]
